# Efficient Heterologous Transformation of *Chlamydomonas reinhardtii npq2* Mutant with the Zeaxanthin Epoxidase Gene Isolated and Characterized from *Chlorella zofingiensis*

**DOI:** 10.3390/md10091955

**Published:** 2012-09-12

**Authors:** Inmaculada Couso, Baldo F. Cordero, María Ángeles Vargas, Herminia Rodríguez

**Affiliations:** Institute of Plant Biochemistry and Photosynthesis, CIC Cartuja, University of Seville and CSIC, Avda. Américo Vespucio no. 49, 41092-Seville, Spain; Email: inmaculada.couso@ibvf.csic.es (I.C.); baldomero@ibvf.csic.es (B.F.C.); avargas@us.es (M.A.V.)

**Keywords:** *Chlorella zofingiensis*, *Chlamydomonas reinhardtii*, zeaxanthin epoxidase, violaxanthin cycle, carotenoids

## Abstract

In the violaxanthin cycle, the violaxanthin de-epoxidase and zeaxanthin epoxidase catalyze the inter-conversion between violaxanthin and zeaxanthin in both plants and green algae. The zeaxanthin epoxidase gene from the green microalga *Chlorella zofingiensis* (*Czzep*) has been isolated. This gene encodes a polypeptide of 596 amino acids. A single copy of *Czzep* has been found in the *C. zofingiensis* genome by Southern blot analysis. qPCR analysis has shown that transcript levels of *Czzep* were increased after zeaxanthin formation under high light conditions. The functionality of *Czzep* gene by heterologous genetic complementation in the *Chlamydomonas* mutant *npq2*, which lacks zeaxanthin epoxidase (ZEP) activity and accumulates zeaxanthin in all conditions, was analyzed. The *Czzep* gene was adequately inserted in the pSI105 vector and expressed in *npq2*. The positive transformants were able to efficiently convert zeaxanthin into violaxanthin, as well as to restore their maximum quantum efficiency of the PSII (Fv/Fm). These results show that *Chlamydomonas* can be an efficient tool for heterologous expression and metabolic engineering for biotechnological applications.

## Abbreviations

gDNA: Genomic DNA
UPGMA: Unweighted Pair Group Method with Arithmetic Mean
PSII: Photosystem II
qPCR: Quantitative Polymerase Chain Reaction

## 1. Introduction

In photosynthetic organisms, carotenoids participate in light harvesting within the reaction centers and the light harvesting complexes (LHCs), the protection of the photosynthetic apparatus via the direct quenching of chlorophyll reactive intermediates and/or singlet oxygen molecules, the dissipation of absorbed energy beyond that required for photosynthesis [1,2], and the structural maintenance of photosynthetic complexes [3]. Therefore, carotenoids participate in keeping a balance between capture, use of light and cell protection of excess excitation energy. In particular the xanthophylls zeaxanthin and lutein, whose formation is enhanced under high light conditions, help in the efficient transition of LHCs of PSII to a conformation that favors the thermal dissipation of parts of the excess of excitation energy. This energy dissipation, measured as non-photochemical quenching of the chlorophyll fluorescence (NPQ) prevents over-reduction of the reaction centers and must be strictly controlled to avoid photosynthetic efficiency loss [4,5]. 

The photo-protective functions of the de-epoxidazed carotenoid zeaxanthin have been intensively studied [6], however, it was not until 1989 when Demming-Adams and co-workers [7] first proposed a now widely accepted role linking zeaxanthin formation with photoprotection within the violaxanthin cycle. The dissipation of excess excitation energy mediated by zeaxanthin in this cycle is a key process in the photosynthetic systems of plants and algae [8,9]. A similar cycle, known as the diadinoxanthin cycle, has also been identified in certain algal groups with cycling occurring between the two xanthophylls diadinoxanthin and diatoxanthin, which only comprises one de-epoxidation step [10,11,12,13]. However, in these algae this cycle is the main one and the role of the violaxanthin cycle is mainly to produce intermediates for the biosynthesis of the diadinoxanthin cycle pigments. 

The violaxanthin cycle is found in plants and the green (chlorophyta) and brown (phaeophyceae) algae. In this cycle, when the irradiance is higher than that required for photosynthesis in the chloroplasts of plants and algae, a reversible violaxanthin de-epoxidation reaction occurs to form the intermediate antheraxanthin, subsequently resulting in the accumulation of zeaxanthin in a second de-epoxidation reaction. The extent of the de-epoxidation depends on two factors at least, namely, the pigment pool size and the fraction of the violaxanthin pool that is available for de-epoxidation [14]. When the absorbed irradiance is lower than that required for the saturation of the photosynthetic apparatus, zeaxanthin is converted back into violaxanthin. This reaction occurs on the stromal side of the chloroplast thylakoid membrane, and it is relatively slower than the de-epoxidation reaction [15,16]. The de-epoxidation sequence of the violaxanthin cycle is catalyzed by the enzyme violaxanthin de-epoxidase (VDE), and the epoxidation from zeaxanthin into violaxanthin is carried out by the enzyme zeaxanthin epoxidase (ZEP). 

ZEP has a specificity for carotenoids with a 3-hydroxy-β-hexenyl ring and requires the presence of NADPH and FAD as cofactors, ferredoxin and ferredoxin-like reducing agents are also required for its activity [17]. The first isolation of this gene was performed in a transposon-tagged *Nicotiana plumbaginifolia* ABA-deficient mutant called *aba2* [18,19] and many genetic studies on this cycle have been performed in plants, such as *Arabidopsis thaliana* in which different *aba* mutants have been characterized, providing an opportunity to study the physiological roles of epoxy-carotenoids in photosynthesis [20], as well as the *npq* mutants in the green alga *Chlamydomonas reinhardtii* [21]. This unicellular green alga has been used as a good alternative molecular system to plants in order to study photosynthesis and other metabolic processes [22]. 

In 1997, Niyogi and co-workers [21] generated a series of violaxanthin cycle mutants in an Arg^-^ auxotroph *Chlamydomonas reinhardtii* strain. These mutants were obtained by insertional mutagenesis using a linearized vector containing the wild type argininosuccinate lyase gene (*arg7*). These mutants exhibited an aberrant NPQ, being defective in two different NPQ phases, either being defective in the quick-turn-over first phase (*npq4*), or in the slower second phase (*npq1*) or in both of them (*npq2* and *npq11*). *Npq2* had lost the zeaxanthin epoxidase activity, since it exhibited a violaxanthin cycle pool of entirely zeaxanthin even after dark adaptation. After examining the co-segregation of the insertional mutagen (ARG7 DNA) and the *npq* aberrant phenotype in the *npq2*, it was concluded that this mutation was closely linked to a single insertion locus of the ARG7 vector. 

The chlorophyta *Chlorella zofingiensis* is considered as a model organism to study the regulation of the carotenoid biosynthetic pathway since it produces both the primary carotenoid lutein as well as the secondary carotenoid astaxanthin [23]. Several carotenogenic genes have been isolated and characterized in this microalga, such as β-carotene oxygenase (*bkt*) [24], phytoene desaturase (*pds*) [25], carotene β-hydroxylase (*chyB*) [26], lycopene β-cyclase (*lcyB*) [27] and phytoene synthase (*psy*) [28]; however, genes involved in the violaxanthin cycle have not been isolated yet. 

In this paper, we report the isolation and characterization of the zeaxanthin epoxidase gene involved in the violaxanthin cycle in *Chlorella zofingiensis*. The transcriptional regulation of this gene in response to light and nitrogen, as well as its functionality by heterologous transformation of *Chlamydomonas reinhardtii npq2* mutant, which lacks ZEP activity, has also been performed. 

## 2. Results

### 2.1. Isolation and Characterization of the *ZEP* Gene and Deduced Protein Sequence of *C. zofingiensis*

**Figure 1:**
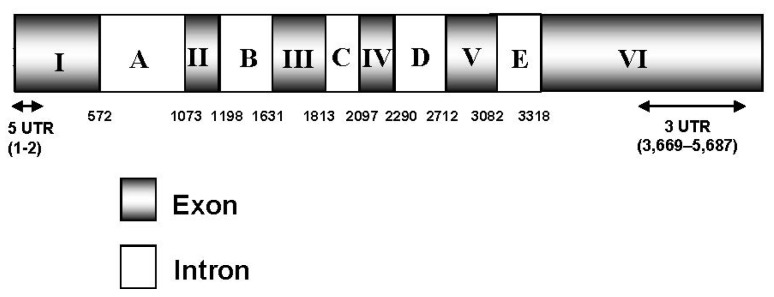
*Czzep* scheme. The diagram shows that the *Czzep* gene consists of six exons (I–VI) and five introns (A–E). The 5′ UTR and 3′ UTR sequences are indicated with arrows and correspond to the positions 1–2 bp and 3669–5687 bp, respectively. Numbers represent the cDNA coordinates (bp). UTR, untranslated region.

Different degenerate primers were designed on the basis of the conserved motifs present in ZEP amino acid sequences from microalgae and plants. A partial *Czzep* cDNA fragment of 1100 bp was isolated by PCR amplification using the degenerate primers ZEP-1F and ZEP-1R (Table 1). A complete basic local alignment search tool (BLAST) homology search in the Genbank database (NCBI) showed that this fragment had enough similarity with the ZEP genes from other species, and provided sequence information for designing specific primers for rapid amplification of 5′ and 3′ cDNA ends (RACE-PCR). This analysis generated a full-length cDNA of 3836 bp, which contained an Open Reading Frame (ORF) of 1791 bp, a very short two nucleotides of 5′ untranslated region (UTR), and a long 3′ UTR of 2043 nucleotides. The predicted protein has 596 amino acid residues, with an estimated molecular weight of 64.50 kDa, a theoretical isoelectric point of 8.31 and an instability index of 29.87 (data obtained with ProtParam program). The differences between the *C. zofingiensis zep* gene and the cDNA sequence were compared and revealed the presence of six exons and five introns (Figure 1).

**Table 1:** Nucleotide sequences of primer pairs used for PCR amplification.

Primer	Sequence (5′→3′)
**Partial ZEP fragment**	
**ZEP-1F**	tggtgggcgccgayggnathhg
**ZEP-1R**	cgcccacgtcggtgswnacraarta
**5′ and 3′ RACE**	
**NGSP-ZEP-5** **′R**	gatcttggaccagataccatcagcacccaca
**GSP-ZEP-5** **′R**	agcatgtatagccgctgtagttggg
**NGSP-ZEP-3** **′F **	cagatttcacgccagcagacattgacat
**GSP-ZEP-3** **′F**	gcggctatacatgctacacagggatct
**Genomic DNA amplification**	
**G-ZEP-1F**	atggggacttcagtacctgca
**G-ZEP-1R**	aacagccagcacaagacctcc
**G-ZEP-2F**	caagaccagaaagcgtcacggc
**G-ZEP-2R**	tgctgcacacctgtgacagggt
**G-ZEP-3F**	gatcctgattgatgccgtggcg
**G-ZEP-3R**	tgctccatagcatctgccagat
**G-ZEP-4F**	atttgggtcagggtggctgcat
**G-ZEP-4R**	ggtgcgaaaagggtatgaagaacg
**G-ZEP-5R**	cgctctacacttggctgtagtg
**G-ZEP-6F**	atcaatggcatggagtacagga
**G-ZEP-6R**	tagcacgatgctgcaagatcacgt
**G-ZEP-7F**	ctggtgtgcagattggttttgccg
**G-ZEP-7R**	Agtattcactcactatgaactgatgcc
**G-ZEP-8F**	ccattgaagatgcttatcagttggcagc
**G-ZEP-8R**	ggcatacatgctctgagcaggcataag
**PCR for *Chamydomonas reinhardtii* transformation** **^a^**	
**pStpZEPxhoF**	cgcctcgagcatggggacttcagtacctgcaaat
**pStpZEPecoR**	ccgaattcgtggctgttatatttgtgctgttg
**CzZEP expression**	
**RT-Czzep-F**	cgaacgtgatcttacagccat
**RT-Czzep-R**	gatacgatctcctgtgatgcagcca
**CBLP expression**	
**RT-cblp-F**	cgccacccagtcctccatcaaga
**RT-cblp-R**	Ctaggcgcggctgggcatttac

F, forward; R, reverse. ^a^ *Xho*I and *Eco*RI restriction sites (lowercase letters underlined) were added for cloning the gene into the corresponding cut sites of the pSI105Tp1 vector.

**Figure 2:**
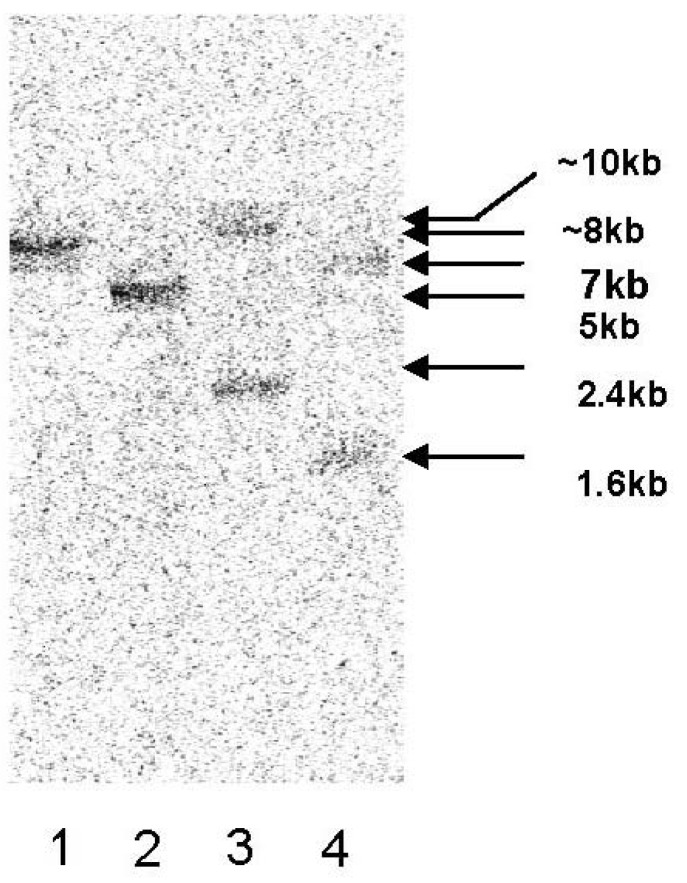
Southern blot analysis of genomic DNA from *C. zofingiensis*. gDNA was digested with ScaI (Lane 1), NdeI (Lane 2), BamHI (Lane 3) or NcoI (Lane 4), electrophoretically separated on a 0.8% agarose gel, blotted and hybridized at high stringency with a probe of 662 bp of the *Czzep* gene amplified by PCR.

For the determination of the copy number of the *zep* gene in the genome of *C. zofingiensis*, genomic DNA was digested with four different restriction enzymes *Sca*I, *Nde*I, *BamH*I and *Nco*I and subjected to Southern blot analysis at different conditions of stringency. Using a 662 bp fragment of *Czzep* as a probe, strong hybridization signals were obtained with the different digestions. The digestion with *Nco*I and *BamH*I enzymes, which cut once inside the probe sequence, showed two bands, while digestion with *Nde*I or *Sca*I, with no restriction site in the probe, exhibited only one band (Figure 2). These results suggest the presence of a single copy of the *zep* gene in the genome of *C. zofingiensis*.

**Figure 3:**
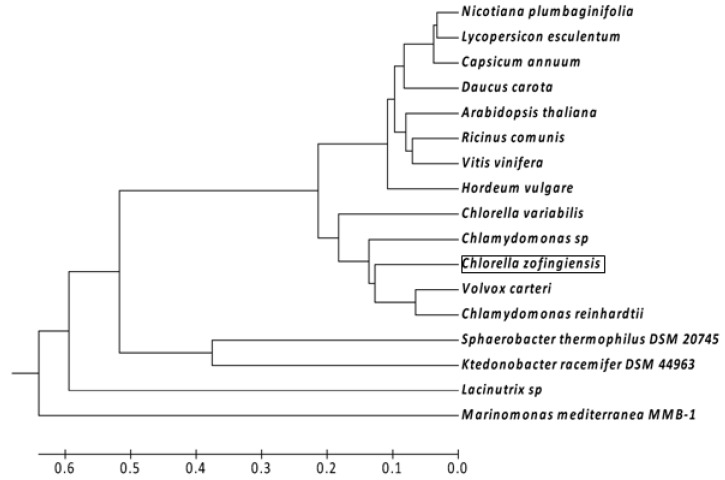
Unweighted pair group method with arithmetic mean (UPGMA) tree analysis of the indicated plant, algal and bacterial ZEP amino acid sequences. The GenBank accession numbers for other species are as follows: *Nicotiana plumbaginifolia* (X95732.1); *Lycopersicon esculentum* (Z83835.1); *Capsicum annuum* (Q96375); *Daucus carota* (ABB52077.1); *Arabidopsis thaliana* (AAG17703); *Ricinus comunis* (XP_002523587.1); *Vitis vinifera* (AAR11195.1); *Hordeum vulgare* (BAK08085.1); *Chlorella variabilis* (EFN52633.1); *Chamydomonas sp* (AAO48941.1); *Volvox carteri* (XP_002953670.1); *Chlamydomonas reinhardtii* (XP_001701701.1); *Sphaerobacter thermophilus* DSM 20745 (ACZ40773.1); *Ktedonobacter racemifer* DSM 44963 (ZP_06974439.1); *Lacinutrix* sp. (AEH02389.1); *Marinomonas mediterranea* MMB-1 (ADZ93016.1). The tree is drawn to scale, with branch lengths in the same units as those of the evolutionary distances used to infer the phylogenetic tree. The evolutionary distances correspond to the number of amino acid substitutions per site and were computed using the Poisson correction method.

The BlastP search results demonstrated that the cloned CzZEP showed the highest overall homology sequence with other ZEP from green algae, such as *Volvox carteri* (identity, 66% and similarity, 77%), *Chlorella variabilis* (identity, 69% and similarity, 79%) and *Chlamydomonas reinhardtii* (identity, 69% and similarity, 80%). The GC content of the *Czzep* coding region was 52.4%, which was lower than that of *V. carteri* (60.5%), *C. variabilis* (68.1%) or of *C. reinhardtii* (68.8%). The phylogenetic analysis of ZEP from green algae, plants and bacteria is illustrated in Figure 3. This analysis was performed in MEGA5 software [29] using the unweighted pair group method with arithmetic mean (UPGMA) method. The predicted CzZEP forms a cluster with the ZEP of the green algae studied, which are phylogenetically close to ZEP of plants (between 60% and 63% of identity and between 73% and 76% similarity). CzZEP was distantly related to bacterial zeaxanthin epoxidases (between 30% and 34% of identity and 45%–50% of similarity). 

Since in microalgae and plants ZEP is located in the chloroplast membranes, we analyzed the CzZEP sequence with different programs to determine both the presence of a signal peptide and transmembrane domains. Target-P and Chloro-P programs identified an *N*-terminal chloroplast transit peptide of 60 aa length. Analysis with TMHMM and TopPred servers identified four deduced transmembrane domains of CzZEP located between amino acids 26–46, 144–164, 502–522 and 549–569. The domain structure analysis with Interpro Scan showed two monooxygenase FAD-binding domains located between amino acids 144–160 and 439–473, as well as four aromatic-ring hydroxylase-like domains located between amino acids 145–167, 308–323, 438–453, and 453–469 (data not shown).

### 2.2. Effect of Irradiance and Nitrogen on the Expression of *Czzep* Gene

**Figure 4:**
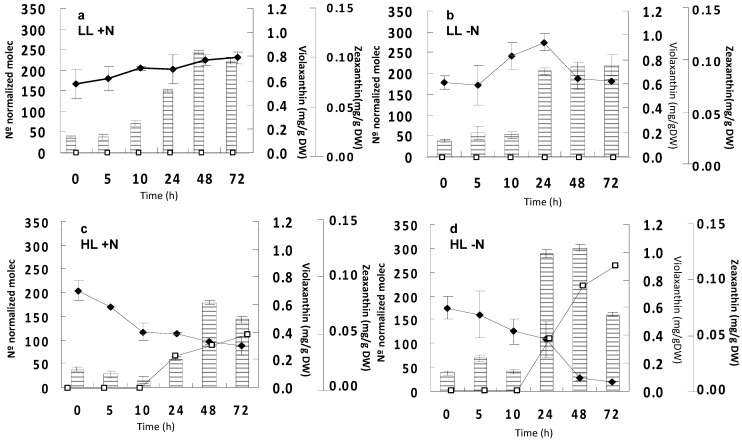
Effect of irradiance and nitrogen availability on the mRNA levels of the zeaxanthin epoxidase gene (*zep*) and cellular content of violaxanthin and zeaxanthin in *Chlorella zofingiensis*. (**a**) Low irradiance (20 µmol photons m^−^^2^ s^−^^1^) and constant nitrate concentration (LL +N); (**b**) low irradiance and nitrate deprivation (LL −N); (**c**) high irradiance (300 µmol photons m^−^^2^ s^−^^1^) and constant nitrate concentration (HL +N); and (**d**) high irradiance and nitrate deprivation (HL −N). Columns indicate mRNA levels of the *zep* gene; (♦), violaxanthin content (mg g^−^^1^ DW); (□), zeaxanthin content (mg g^−^^1^ DW). Error bars indicate the standard deviations of four independent measurements. DW, dry weight.

*C. zofingiensis* cells were grown photoautotrophically at low irradiance and constant nitrate, as indicated in the Experimental Section, until the middle of the exponential phase. Then, cells were kept in the dark for 18 h, in order to make the transcript levels come down to basal values. After this dark period, cells were subjected to either low or high irradiance, under either constant nitrogen or nitrogen-deprivation conditions. The evolution with time of mRNA levels of the *Czzep* gene as affected by irradiance and nitrogen availability was monitored by quantitative real time PCR (qRT-PCR). In addition, changes in zeaxanthin and violaxanthin cellular contents were also determined in order to correlate mRNA levels with the biosynthesis of these carotenoids. As shown in Figure 4a,b, at low irradiance the levels of transcripts did not start to increase until 24 h after the dark period, regardless of nitrate availability. Under high irradiance and constant nitrogen conditions, the transcript levels of *Czzep* decreased between 10% and 60% after 5 h and 10 h from the beginning of the experiment, respectively (Figure 4c). Although there was also a high increase of the transcripts levels after 24 h under these conditions, they did not reach the levels registered at low irradiance. However, an additive response was detected in the nitrogen-free culture under high light conditions (Figure 4d), transcript levels after 24 h of induction being higher than those exhibited under low irradiance. With regards to the cellular carotenoid content, zeaxanthin was only registered at high irradiance, the levels of this carotenoid increased with time after 10 h, with a much higher accumulation of this carotenoid taking place under nitrogen depletion conditions (Figure 4c,d). Antheraxanthin was only detected at high irradiance as well, its levels ranging from 0.12 mg∙g^−1^ DW at 10 h to 0.09 mg g^−1^ DW at 72 h of high light exposure. Both zeaxanthin and antheraxanthin contents seem to be correlated with the increased mRNA levels of *Czzep*. The concentration of violaxanthin decreased with time at high irradiance, especially in the case of nitrogen-free conditions, while in the case of low light conditions it remained almost constant and only a little increase was detected at the 10 h or 24 h under nitrogen deprivation. Under high irradiance, astaxanthin was also present both in nitrogen depleted and in nitrogen repleted conditions, reaching values of 0.21 mg g^−^^1^ DW and 0.5 mg g^−^^1^ DW after 48 h, respectively. 

### 2.3. Functional Analysis of the ZEP from *C. zofingiensis* by Heterologous Genetic Complementation in *C. reinhardtii npq2* Mutant

#### 2.3.1. Transformation of *C. reinhardtii npq2* Mutant with *zep* Gene from *C. zofingiensis* and Screening of the Obtained Transformants

**Figure 5:**
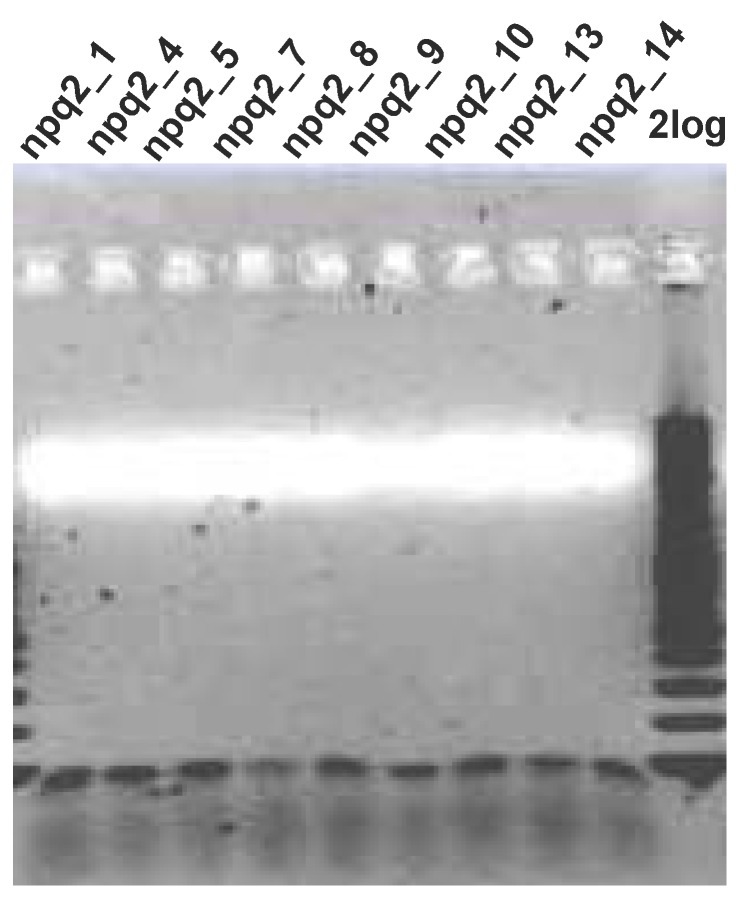
Verification of the plasmid pSI105-Tp1-*Czzep* insertion in the genome of *C. reinhardtii* by PCR. *C. reinhardtii npq2* cells transformed with the plasmid pSI105-Tp1-*Czzep* were grown in TAP medium with paromomycin (30 µg mL^−1^), and paromomycin-resistant colonies were tested by PCR. Lane 10 is the 2log DNA ladder (0.1–10 kb, Biolabs). Lanes 1–9 correspond to some analyzed transformants that were found to be positive.

Since the use of *E. coli* (DH5α) engineered to produce zeaxanthin did not allow the functional characterization of the *Czzep* gene, probably due to the lack of cofactors necessary for enzymatic activity, the functionality of this gene was assayed by heterologous genetic complementation in the *C. reinhardtii npq2* mutant. For this purpose, the complete coding region of *Czzep* was amplified by PCR and cloned between the *Xho*I and *EcoR*I restriction sites of the *Chlamydomonas* expression vector pSI105-Tp1, resulting in the plasmid pSI105-Tp1-zep. *C. reinhardtii npq2* mutant cells were transformed with this plasmid, and the transformants were *a priori* selected on the basis of their paromomycin resistance. The colonies obtained were screened for the insertion of *Czzep* cDNA in their genome by PCR. Figure 5 shows some of the positive transformants analyzed exhibiting a band of approximately 0.1 kb, which corresponds to the *Czzep* cDNA integrated in their genome. The primers used for PCR analysis to confirm this integration were G-ZEP-2F and G-ZEP-1R (Table 1) with a final amplification of 75 bp. More than 150 colonies resistant to paromomycin were analyzed and 113 of them (≈75%) were found to be positive. 

#### 2.3.2. Analysis of Carotenoid Content, Maximum Quantum Efficiency and mRNA Level of *Czzep* Gene in Both *npq2* Mutant of *C. reinhardtii* and *Czzep*-Transformed Strains of *npq2* Mutant

The mRNA relative abundance of *Czzep* gene of *C. reinhardtii npq2 * mutant, which lacks ZEP activity and accumulates zeaxanthin in all conditions, and *Czzep*-transformant strains of *npq2* mutant was monitored by qRT-PCR, and changes in cellular carotenoids content were determined in order to correlate transcript levels with the biosynthesis of specific carotenoids. In addition, the photosynthetic capacity of *npq2* mutant and the transformants was also determined by measuring their maximum quantum yield (Fv/Fm).

**Figure 6:**
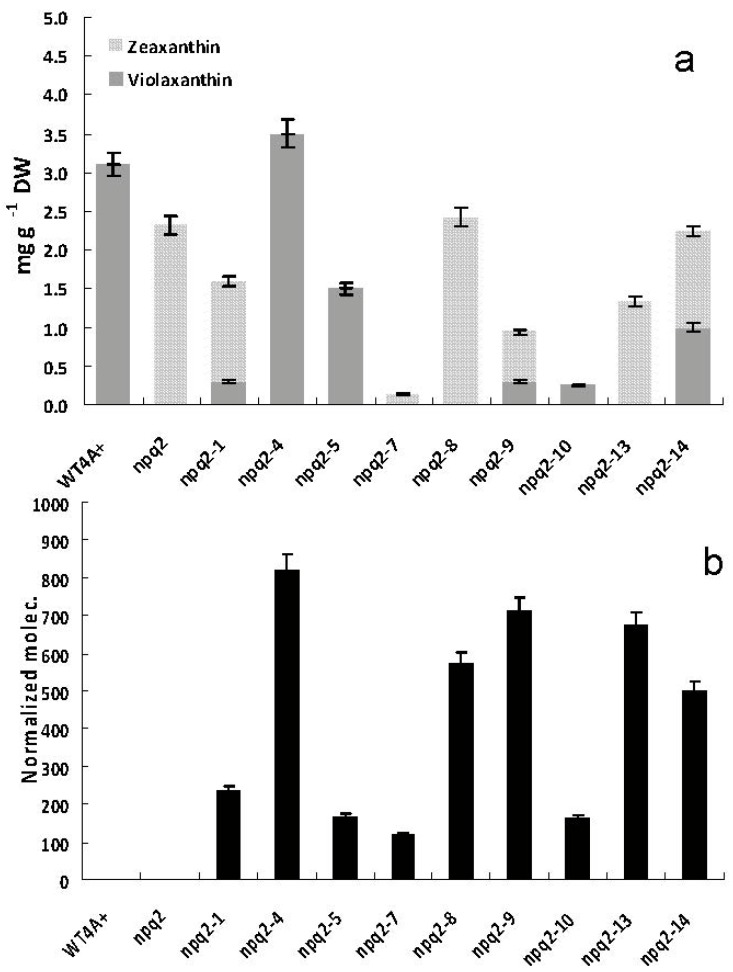
Zeaxanthin and violaxanthin content (**a**) and mRNA relative abundance of *Czzep* (**b**) in cells of *C. reinhardtii npq2* mutant (*npq2*) and nine selected transformants (*npq2-1*, *npq2-4*, *npq2-5*, *npq2-7*, *npq2-8*, *npq2-9*, *npq2-10*, *npq2-13* and *npq2-14*). **a**, Zeaxanthin (grey bar) and violaxanthin (black bar); **b**, Levels of *Czzep* transcripts were normalized in respect to the housekeeping control gene (*cblp*). Error bars indicate the standard deviations of four independent measurements.

Lutein and β-carotene were the major carotenoids both in *npq2* mutant and *Czzep* transformant cells, the levels of those carotenoids being higher in *Czzep* transformants (ranging from 1.67 to 2.29 mg g∙DW^−1^ in the case of lutein and 0.78 to 1.19 mg g∙DW^−1^ in the case of β-carotene) than in *npq2* mutant (0.55 mg g∙DW^−1^ in the case of lutein and 0.41 mg g∙DW^−1^ in the case of β-carotene). Thus, the *npq2-4* transformant showed a content of lutein and β-carotene six- and four-fold higher, respectively, than the mutant. Most of the transformants (approximately 80% of the selected positives) exhibited a lutein and β-carotene contents of 2.8- to 3.2- and 1.8- to 2.5-fold higher than the *npq2* mutant, respectively. The violaxanthin and zeaxanthin contents and the relative mRNA levels of the *Czzep* gene in the *npq2* mutant and nine selected *Czzep npq2* transformants are illustrated in Figure 6. Most of the transformants analyzed showed a restored zeaxanthin epoxidase activity, some of them exhibiting a total conversion of zeaxanthin into violaxanthin, such as *npq2-4*, which seems also to exhibit the highest expression pattern of the *Czzep* gene. Some transformants, however, did not show a total conversion of zeaxanthin into violaxanthin or a restored zeaxanthin epoxidase activity, although in all of them the Cz*zep* gene was adequately transcribed. Nevertheless, the expression levels of *Czzep* in transformants were rather heterogeneous coinciding with the differences found in the carotenoid levels. In fact, the highest *Czzep* mRNA level was found in the *npq2-4* transformant, which reached about seven-fold the levels of the transformants *npq2-7*, *npq2-5* and *npq2-10*. This correlated with the highest level of carotenoids shown by *npq2-4* transformant. *Npq2-9* and *npq2-13* transformants also showed a high level of *Czzep* mRNA, reaching about five times the level of the lowest yielding transformants. Therefore, the functional characterization of the recently isolated *Czzep* gene could be achieved by expression of this gene in the transformants and the restoration of zeaxanthin epoxidase activity. 

The Fv/Fm parameter is widely considered to be a sensitive indicator of plant photosynthetic performance. In this sense, we wanted to determine why lacking a functional violaxanthin cycle *npq2* mutant could determine a lower photosynthetic capacity of the *npq2* mutant. At the same time, we also wanted to measure the differences, if they do exist, in the maximum quantum yields (Fv/Fm) among the different transformants and the parental strain. In Table 2, it is shown that the Fv/Fm value of the *npq2* mutant is 25% lower than that of the Wild type. In addition, Fv/Fm values of the transformants were restored by the exogenous CzZEP activity from 84.6% for *npq2-5* to 100% for *npq2-4*. The full recovery of the Fv/Fm value shown by the *npq2-4* transformant was correlated with the fact that this transformant was able to completely transform the zeaxanthin pool into violaxanthin.

**Table 2:** Maximum quantum yield data and the percentages recovered in the transformants analyzed.

Strains	Fv/Fm	% Fv/Fm recovered
**WT4A+**	0.65 ± 0.014	---
***npq2***	0.485 ± 0.003	---
***npq2-1***	0.585 ± 0.017	89.2%
***npq2-4***	0.65 ± 0.014	100%
***npq2-5***	0.555 ± 0.003	84.6%
***npq2-7***	0.585 ± 0.007	89.2%
***npq2-8***	0.6 ± 0.007	92.3%
***npq2-9***	0.585 ± 0	89.2%
***npq2-10***	0.605 ± 0.003	93.1%
***npq2-13***	0.58 ± 0	89.2%
***npq2-14***	0.61 ± 0.007	93.8%

## 3. Discussion

Although previous work concerning zeaxanthin epoxidase has essentially focused on its role in ABA biosynthesis during drought stress and seed development in plants [19,20,30], its relationship with photosynthesis and the carotenoids synthesis pathway using mutants lacking the zeaxanthin epoxidase activity has also been reported both in plants and algae [21,31,32,33,34]. The *zep* gene cDNA has been isolated from higher plants such as *Nicotiana plumbaginifolia* (tobacco), *Capsicum annuum* (pepper), *Lycopersicon esculentum* (tomato) and *Arabidopsis thaliana* [15,18,34] and from some algae such as the well-known *Chlamydomonas reinhardtii* [35]. In this work, we describe the isolation and characterization of the *zep* gene from the green alga *Chlorella zofingiensis*. This new gene showed six exons and five introns, and the ORF of the cDNA encoded a hypothetical protein of 596 amino acids (Figure 1). The length of the putative protein was similar to those described for microalgae, being slightly shorter than plant enzymes, according to alignments performed with the Blocks program (data not shown). Southern blot analysis has demonstrated that in *C. zofingiensis*, as in other green algae such as *C. reinhardtii* [35] and *Chlorella variabilis* [36], as well as in angiosperms, only a single gene encoding ZEP is present. However, it has been recently described that other algae classes such as the diatoms *P. tricornutum* and *T. pseudonana* contain three and two copies, respectively, of the *zep* gene, which could be a reflection of the presence of both cycles, violaxanthin cycle and diadinoxanthin cycle in these organisms. The diadinoxanthin cycle is generally found in heterokons but it is absent in plants and chlorophytes [37]. On the other hand, the *zep* gene has not been found in the genomes of cyanobacteria or the red algae, such as *C. merolae* and *G. sulphuraria* [38].

The first *zep* gene isolated in *Nicotiana plumbaginifolia* [18], called *aba2*, encoded a chloroplast-imported flavin monooxygenase (FMO) containing a FAD-binding domain. The amino acid sequences of the pepper [15], tomato [34] and tobacco ZEP were similar to that of a flavoprotein monooxygenase (salicylate-1-monooxygenase) and those of a number of other bacterial hydroxylases [39]. Therefore, ZEP enzymes are considered FAD-dependent monooxygenases. The amino acid sequence analysis of the predicted protein encoded by the new gene isolated from *C. zofingiensis* revealed two monooxygenase FAD-binding domains [37,40]. In addition, the predicted CzZEP sequence showed four putative transmembrane domains that could play a role in the localization of this enzyme on the stromal side of the chloroplast thylakoid membrane, and a transit peptide which would allow CzZEP to travel from the cytoplasm, where the polypeptide is synthesized, to its final location in the chloroplast as in the case of other carotenogenic enzymes in this alga which are encoded by nuclear genes [23,27].

Different environmental conditions can show different regulations of the carotenoid synthesis pathway. As an example, either low light intensities or darkness stimulate the backward reaction of the violaxanthin cycle resulting in the conversion of zeaxanthin back to violaxanthin in *Arabidopsis* and *C. reinhardtii* [41,42]. In photoautotrophically grown *C. zofingiensis* cultures, it is known that the combination of both high irradiance and nitrogen starvation causes changes in the cellular content of several carotenoids [43,44,45]. Recently, it has been shown that high irradiance in this alga up-regulates the *pds*, *chyB*, and *bkt* genes, significantly enhancing astaxanthin synthesis [25,26]. In addition, the nitrogen deprivation *per se* up-regulated the *lcyB* gene, but did not trigger astaxanthin biosynthesis [27]. We have studied the regulation of the *Czzep* gene by nitrogen and irradiance, determining the mRNA levels as well as the cellular content of violaxanthin and zeaxanthin (Figure 4). The transcript levels of *Czzep* did not increase until 24 h under low irradiance conditions, coming from a dark period, regardless of the nitrogen availability, without any accumulation of zeaxanthin and with the violaxanthin content remaining virtually constant. Therefore, it is possible that nitrogen deprivation itself could not affect the level of *zep* transcripts. Under high irradiance and as previously described, the light activated the de-epoxidation of violaxanthin, registering zeaxanthin production after 10 h of exposure and the decrease of the violaxanthin content, the effect being more pronounced under nitrogen deprivation (about two-fold zeaxanthin level of the nitrogen complete culture), showing a possible additive effect of those two stress conditions. However, mRNA levels of *Czzep* surprisingly increased after 10 h, reaching the highest values under nitrogen starvation conditions. For both high light and high light combined with nitrogen starvation stresses, the increase in zeaxanthin content was lower than the decrease in violaxanthin content. This could be due to the presence of antheraxanthin as well as to the astaxanthin accumulation, since in *C. zofingiensis* it has been reported that astaxanthin is mainly synthesized from zeaxanthin instead of β-carotene as in *H. pluvialis* [46]. 

The high violaxanthin de-epoxidase activity under high irradiance has been described previously as being due to a result of the presence of a transmembrane proton gradient during periods of high irradiance [14,47,48]. Until now, the regulation of the ZEP is still not well understood. Some authors have proposed that the zeaxanthin epoxidase activity seems to be constitutive [49], as this is not regulated by the stromal pH. Since ZEP requires molecular oxygen as a second substrate and NADPH as a cofactor, both factors might principally control or limit ZEP activity [49]. The predicted protein encoded by the new gene isolated from *C. zofingiensis* showed a high homology degree with ZEP of plants and algae (Figure 3). This could be sufficient to consider this new gene as a zeaxanthin epoxidase, but the functional analysis has confirmed this hypothesis. By complementation in *E. coli* using the pAC25crtx plasmid, which [50] contains the carotenogenic genes responsible for the formation synthesis of zeaxanthin, together with the *Czzep* gene cloned into pQE80-L expression vector, no conversion of zeaxanthin into violaxanthin or the intermediate antheraxanthin was detected. Molecular oxygen (O_2_), NADPH and reduced ferredoxin are known to be co-substrates for the epoxidation reaction [15,17,51]. The prokaryotic host *E. coli* cannot provide reduced ferredoxin and this seems to be the reason why some *zep* genes do not work in this bacterium, as it was also the case of *Nicotiana plumbaginifolia* [18,52]. In *Chlamydomonas reinhardtii*, ZEP functionality has been studied by using forward genetics. The *Chlamydomonas npq2* mutant was isolated because it exhibited an unusual violaxanthin cycle pool, consisting entirely of zeaxanthin in all conditions. In contrast to other *npq* mutations, *npq2* phenotype co-segregated with arginine producer phenotype, demonstrating that the mutation affects a single gene encoding the zeaxanthin epoxidase enzyme [21]. As the CzZEP functionality could not be performed in *E. coli*, the *Czzep* gene was inserted into a *Chlamydomonas* expression vector to be heterologously expressed *in vivo* in the *Chlamydomonas npq2* mutant. The transformants obtained were able to convert back zeaxanthin into violaxanthin, which confirmed the functionality of the *Czzep* gene isolated. 

At the moment, *Chlamydomonas* ZEP is the only zeaxanthin epoxidase enzyme whose functionality has been reported in algae [21,53]. On the other hand, the complementation of the mutation that *npq2* carried by *Czzep*, supports the idea that *Chlamydomonas* can be an efficient host for expressing exogenous genes. 

Several studies about over-expression of the zeaxanthin epoxidase gene have been reported in plants [54,55]. Transgenic lines in tomatoes shown altered de-epoxidation kinetics of the violaxanthin cycle especially under stress conditions, but nothing was described regarding the concentration of the rest of carotenoids found in their profile. In the case of the CzZEP transformants of *npq2* mutant obtained we found higher levels of β-carotene and lutein reaching between three to six times the levels of the *npq2* control cells. Moreover, these increments in carotenoids also correspond to increased *Czzep* mRNA levels of the transformants analyzed. Thus, the *npq2-4* transformant, which exhibited the highest expression pattern, also showed the highest carotenoids levels (Figure 6a) as well as a total conversion from zeaxanthin into violaxanthin (Figure 6b). *Chlamydomonas* transformants over-expressing the phytoene synthase gene of *C. zofingiensis* isolated also by us, exhibited a carotenoids content up to 2.2-fold the level of the control cultures [23]. Phytoene synthase catalyzes the first step of the carotenoid biosynthetic pathway and it is considered as a rate-limiting key enzyme in this pathway. Since the *Czzep* transformants isolated in this work showed higher carotenoids levels than parental ones, the violaxanthin cycle can be postulated as a key step to be considered for biotechnological applications and genetic manipulation. 

It has been shown that the violaxanthin cycle pigments are bound to light-harvesting complexes of the two photosystems [56,57]. In fact, ZEP has been proposed to be located close to the light harvesting complex of PSII (LHCII) to enable the binding of violaxanthin and the intermediate antheraxanthin to the antenna complex [58]. The role of zeaxanthin in the chlorophyll fluorescence quenching has also been largely discussed, concluding that Lhcb proteins replace violaxanthin by zeaxanthin in the L2 site which induces the quenching of the chlorophyll fluorescence by increasing the relative amplitude of the short fluorescence lifetime component found in these Lhcb proteins. This correlates with energy dissipation and regulation of the energy transfer to the PSI reaction centers [59,60]. Kalituho and co-workers in 2007 [61] reported a reduced electron transport in *Arabidopsis npq2* mutant due to sustained energy dissipation by the high levels of zeaxanthin, and how it might reflect the less efficient utilization of absorbed light energy measured as the maximum quantum yield (Fv/Fm). In the same way, *Chlamydomoans npq2* mutant Fv/Fm values were 25% lower than those in the wild type strain. However, the obtained transformants showed restored Fv/Fm values, recovering the levels of the *Chlamydomonas* wild type cells almost completely (Table 2). These results were also correlated to those obtained from the transformants, some of which showed high expression levels and total conversion from zeaxanthin into violaxanthin. As an example, the *npq2-4* transformant exhibited a 100% restored Fv/Fm, in addition to a higher concentration of carotenoids and mRNA levels of *Czzep*.

## 4. Experimental Section

### 4.1. Strains and Culture Conditions

*Chlorella zofingiensis* SAG 211-14 strain was obtained from the Culture Collection of Göttingen University (SAG, Germany). This green microalga was grown photoautotrophically in Arnon medium [62] modified to contain 4 mM K_2_HPO_4_ and 20 mM NaNO_3_, at 25 °C under continuous illumination (20 μmol photons m^−2^ s^−1^). The light intensity was measured at the surface of the flasks using a LI-COR quantum sensor (model L1-1905B, Li-Cor, Inc. Lincoln, NE, USA). The liquid cultures were continuously bubbled with air supplemented with 1% (v/v) CO_2_ as the only source of carbon. In order to analyze *zep* gene expression, cells were grown in Roux flasks of 1 L capacity laterally and continuously illuminated with mercury halide lamps at either 20 or 300 µmol photons m^−2^ s^−1^ either in the presence or in the absence of nitrate. The nitrate concentration was measured daily and the nitrate consumed by the cells was added to the cultures in order to keep the nitrate constant in the medium.

*Chlamydomonas reinhardtii* WT4a+ (CC-4051) and *npq2* (CC-4101) strains were obtained from the *Chlamydomonas* center’s collection and cultured mixotrophically in either liquid or agar solidified Tris-acetate phosphate (TAP) medium [63] at 25 °C under a continuous irradiance of 20 μmol photons m^−2^ s^−1^. For the analysis of transformants, cells were grown in Erlenmeyer flasks of 100 mL capacity at 25 °C under continuous illumination (20 μmol photons m^−2^ s^−1^) in liquid TAP medium. 

*Escherichia coli* DH5α strain was used as a host for DNA manipulation. 

### 4.2. Genomic DNA and RNA Isolation and cDNA Preparation

DNA and total RNA were isolated using DNeasy Plant Mini Kit and RNeasy Plant Mini Kit (Qiagen, Düsseldorf, Germany), respectively. For PCR screening of *npq2* transformants, a loopful of cells was scrapped from a plate and resuspended in 150 μL of cold distilled water and 350 μL of a buffered solution containing 50 mM Tris-HCl, pH 8, 0.3 M NaCl, 5 mM EDTA, and 2% SDS. The genomic DNA isolation was performed by phenol-chloroform-isoamyl alcohol (50:48:2) extraction and selective precipitation with ethanol, according to previously described protocols [64]. Regarding the quantitative real-time PCR analysis (qRT-PCR), first-strand cDNA synthesis was obtained from total RNA treated with DNase as recommended by the manufacturer, by using the SuperScript First-Strand Synthesis System (Invitrogen, Barcelona, Spain) primed with oligo(dT)_18_ according to the manufacturer’s instructions.

### 4.3. Cloning of *C. zofingiensis zep* cDNA and Genomic Gene

Amino acid sequences deduced from previously cloned *zep* genes from different kinds of algae and plants were aligned for the isolation of the cDNA clone coding the *C. zofingiensis* ZEP. Highly conserved regions were identified, and different pairs of degenerated primers were designed. The PCR products were cloned into pGEM-T vector (Promega, Madison, WI, USA) according to the manufacturer’s manual and then sequenced. The cDNA fragment obtained corresponding to partial *zep* clone analyzed provided sequence information for the designing of gene specific primers for the amplification of 5′ and 3′ cDNA ends by RACE-PCR. All reactions were performed with kits according to the manufacturer’s instructions (Smart RACE cDNA Amplification Kit, Clontech, Mountain View, CA, USA). 5′ and 3′ RACE products were cloned into pGEM-T vector and sequenced. Specific primers were synthesized for genomic DNA amplification based on cDNA sequence. The primer sets used in this study are listed in Table 1. 

### 4.4. Nucleotide Sequence Accession Numbers

The *Czzep* cDNA and genomic DNA sequences have been registered in the EMBL database (EMBL, HE863825 and EMBL, HE863826, respectively).

### 4.5. Sequencing and Phylogenetic Analysis

The deduced amino acid sequence of the *C. zofingiensis* ZEP was compared with other ZEP sequences of algae, plants and bacteria. The sequence analysis and alignments were performed using CLUSTAL_W software. The construction of a phylogenetic tree was performed in MEGA5 [29] using the UPGMA method. The deduced amino acid sequence was subjected to the ProtParam application at the ExPASy server [65] for physical and chemical parameters, Target_P program [66] for the prediction of possible plastid localization, and ChloroP1.1 server [67] for the identification of a chloroplast transit peptide. For transmembrane domains analysis, TMHMM [68] and TopPred [69] servers were used. The domain structure of the predicted protein was analyzed by Interpro Scan [70].

### 4.6. Southern Blot Analysis

Genomic DNA was digested with *Sca*I, *Nde*I, *BamH*I and *Nco*I. The first two enzymes showed one recognition site inside the probed region of the *zep* gene, while the other two did not have any. The probe was prepared by amplifying the amplification of the genomic DNA with primers G-ZEP-4F and G-ZEP-4R, resulting in a 662 bp fragment of *Czzep* gene. The digested DNA was transferred to a Hybond-N membrane (GE Healthcare, Little Chanfont, UK) by capillary transfer and hybridized with the ^32^P labeled DNA probe at both low and high stringency overnight. After hybridization, the radioactivity of the membrane was monitored by the Cyclone Phosphor System (Perkin-Elmer, Waltham, MA, USA).

### 4.7. Chlamydomonas Nuclear Transformation

The complete coding region of *Czzep* was amplified by PCR with primers pStpZEPxhoF and pStpZEPecoR, which were designed to contain *Xho*I and *EcoR*I restriction sites respectively, and cloned into pGEM-T vector (Promega), which carries ampicillin resistance. The PCR product was digested with *Xho*I and *EcoR*I and sub-cloned into the pSI105-Tp1 vector, resulting in the plasmid pSI105-Tp1-zep that was used to transform *Chlamydomonas* cells. The plasmid pSI105-Tp1 is based on the plasmid pSI104-PLK [71], derived from the pSI103 [72], in which the *aphVIII* gene from *Streptomyces rimosus*, encoding an aminoglycoside 3′ phosphotransferase that confers resistance to the antibiotic paromomycin, is expressed under the control of the strong constitutive promoters *rbcS2* and *hsp70A* and terminated by the 3′ untranslated region of *rbcS2*. The construction pSI105-Tp1 also carries a second expression cassette driven by the same two constitutive promoters and terminator region. This second cassette carries the transit peptide of RuBisCO small subunit (*rbcS2*) to target the final peptide into the chloroplast stroma and also carries a polilynker region in which the cDNA from *C. zofingiensis zep* was subcloned in frame with the transit peptide sequence.

Nuclear transformation was carried out using the glass beads method of Kindle (1990) with some modifications [71]. *Chlamydomonas npq2* cells were grown to about 10^7^ cells mL^−1^ then cells were harvested by centrifugation and resuspended in fresh TAP medium to obtain a 100-fold concentrated cell suspension. The concentrated cell suspension (0.6 mL) was added to a conical tube containing 0.3 g of sterile glass beads (Ø 0.4–0.6 mm), 0.2 mL of 20% polyethylene glycol (MW8000) and 1 μg of the desired plasmid. Cells were vortexed and resuspended in 50 mL of fresh sterile TAP medium where they were incubated in the dark overnight. After this incubation, cells were centrifuged and spread onto solid TAP medium with paromomycin (30 μg mL^−1^). Transformed colonies were visible after 4 to 5 days.

### 4.8. Quantitative RT-PCR

The mRNA relative abundance of *C. zofingiensis zep* was examined by qRT-PCR on an IQ5 Real-Time PCR Detection System (BioRad, Hercules, CA, USA), according to [27]. In each experiment, a series of standard dilutions containing a specific concentration of a PCR fragment was amplified in 20 μL of reaction solution containing 1 × SYBR Green PCR Master Mix (Quantimix Easy SYG kit, BioTools B & M Labs, Madrid, Spain) and corresponding primers for *zep* from *C. zofingiensis* (Table 1). After heating at 95 °C for 10 min, the cycling parameters were: fourty cycles of 95 °C for 30 s, 60 °C for 30 s, and 72 °C for 30 s. Finally, the specificity of the qRT-PCR products was confirmed by performing a melting temperature analysis at temperatures ranging from 55 °C to 95 °C at 0.5 °C/min and also by electrophoresis on a 2% agarose gel. Data were captured as amplification plots. Transcription levels of the target gene were calculated from the threshold cycle by interpolation from the standard curve. To standardize the results, the relative abundance of *cblp* gene, which encodes a G-protein β-subunit-like polypeptide [73], was also determined and used as the internal standard in the case of *Chlamydomonas* transformants. The actin gene was also used as the internal standard [74,75] for *zep* mRNA analysis in *C. zofingiensis*. The complete experiments (RNA isolation, cDNA synthesis followed with qRT-PCR) were repeated twice independently, and data are the average of at least three replicates.

### 4.9. Analytical Methods

#### 4.9.1. Cell Concentration and Dry Weight Determinations

The cell number was determined with a Neubauer hemocytometer. For dry weight measurements, aliquots (5 mL) of the cell culture were filtered through Whatman GF/C paper (Whatman plc, Kent, UK), washed three times with ammonium formate 1 M solution, and dried at 80 °C for 24 h.

#### 4.9.2. Carotenoid Extraction and HPLC Analysis

Total pigments were extracted with 80% of acetone (v/v) according to León *et al.* (2005) [76]. Then the samples were centrifuged and analyzed by HPLC using a Waters Spherisorb ODS2 column (4.6 × 250 mm, 5 μm particle size) (Waters, Mildford, MA, USA). The chromatographic method described by [27] was used. Pigments were eluted at a flow rate of 1.0 mL min^−1^ and were detected at 440 nm using a Waters 2996 photodiode-array detector. Identification of carotenoids was achieved by comparison of the individual characteristic absorption spectrum and the retention time with known standards. Quantification was performed using a calibration curve generated with commercially available carotenoids standards from Sigma-Aldrich (St. Louis, MO, USA) and DHI (Holsholm, Germany). 

#### 4.9.3. Photochemical Efficiency Analysis (Fv/Fm Measurements)

Fv/Fm is a parameter widely used to indicate the maximum quantum efficiency of Photosystem II. Fv/Fm is presented as a ratio of variable fluorescence (Fv) over the maximum fluorescence value (Fm). For these measurements, samples were taken from *npq2* transformants obtained and from control cultures in Erlenmeyer flasks at 25 °C under continuous illumination (20 μmol photons m^−2^ s^−1^) and agitation. They were firstly adapted to the dark for 15 min and then these measurements were obtained using 2 mL of culture in an AquaPen-C-AP100 (Photon Systems Instruments, Drasov, Czech Republic). The experiments were performed in duplicate and the given data are the resulting averages of three replicates of each sample. 

## 5. Conclusions

Until now, *Chlamydomonas* ZEP was the only zeaxanthin epoxidase enzyme which functionality was reported in algae. *Czzep* isolation and characterization was performed in this study. The recovered transformants able to convert back zeaxanthin into violaxanthin confirm the functionality of the *Czzep* gene isolated and support the idea that *Chlamydomonas* can be an efficient host for checking the functionality of exogenous genes. Our data on the characterization and regulation of this gene by light and nitrogen could contribute to the understanding of the regulatory mechanism of biosynthesis of carotenoids at the molecular level, which can be very useful for the optimization of the physiological conditions for higher carotenoid production by *C. zofingiensis*.
